# Acceleration of small bowel motility after oral administration of dai-kenchu-to (TJ-100) assessed by cine magnetic resonance imaging

**DOI:** 10.1371/journal.pone.0191044

**Published:** 2018-01-10

**Authors:** Akitoshi Inoue, Akira Furukawa, Hiroshi Yamamoto, Shinichi Ohta, Nguyen Dai Hung Linh, Tulyeubai Syerikjan, Sachiko Kaida, Tsuyoshi Yamaguchi, Satoshi Murata, Toru Obata, Masaji Tani, Kiyoshi Murata

**Affiliations:** 1 Department of Radiology, Shiga University of Medical Science, Otsu, Shiga, Japan; 2 Department of Radiological Science, Tokyo Metropolitan University, Arakawa, Tokyo, Japan; 3 Department of Surgery, Shiga University of Medical Science, Otsu, Shiga, Japan; National Institute of Radiological Sciences, QST, JAPAN

## Abstract

Dai-kenchu-to (TJ-100) is an herbal medicine used to shorten the duration of intestinal transit by accelerating intestinal movement. However, intestinal movement in itself has not been evaluated in healthy volunteers using radiography, fluoroscopy, and radioisotopes because of exposure to ionizing radiation. The purpose of this study was to evaluate the effect of TJ-100 on intestinal motility using cinematic magnetic resonance imaging (cine MRI) with a steady-state free precession sequence. Ten healthy male volunteers received 5 g of either TJ-100 or lactose without disclosure of the identity of the substance. Each volunteer underwent two MRI examinations after taking the substances (TJ-100 and lactose) on separate days. They drank 1200 mL of tap water and underwent cine MRI after 10 min. A steady-state free precession sequence was used for imaging, which was performed thrice at 0, 10, 20, 30, 40, and 50 min. The bowel contraction frequency and distention score were assessed. Wilcoxon signed-rank test was used, and differences were considered significant at a *P*-value <0.05. The bowel contraction frequency tended to be greater in the TJ-100 group and was significantly different in the ileum at 20 (TJ-100, 8.95 ± 2.88; lactose, 4.80 ± 2.92; *P* < 0.05) and 50 min (TJ-100, 9.45 ± 4.49; lactose, 4.45 ± 2.65; *P* < 0.05) between the groups. No significant differences were observed in the bowel distention scores. Cine MRI demonstrated that TJ-100 activated intestinal motility without dependence on ileum distention.

## Introduction

Dai-kenchu-to (TJ-100) is an herbal medicine used to shorten the duration of intestinal transit by accelerating intestinal movement. TJ-100 is routinely used for postoperative ileus, prevention of adhesive bowel obstruction after abdominal surgery, and prevention of various gastrointestinal symptoms, including functional gastrointestinal disorders, especially abdominal distention, weak intestinal peristalsis, and constipation [1, 2]. Scientific and clinical research has been performed to elucidate the mechanism and establish the clinical roles of herbal medicine.

Previous studies have shown that three major mechanisms enhanced intestinal motility, and it has been shown that TJ-100 has an acetylcholine-releasing action in the smooth muscle tissues of the ileum and that 5-HT_3_ and 5-HT_4_ receptors were involved in this action [3, 4]. The administration of TJ-100 was found to significantly increase motilin levels, which improved intestinal motility [5]. TJ-100 acts on transient receptor potential V1 (TRPV1), which is distributed in the sensory nerve and is a channel opened by a nociceptive stimulus, and results in the release of substance P that influences bowel contraction [6]. Furthermore, TJ-100 can increase intestinal blood flow. TJ-100 is an agonist for transit receptor potential A1 (TRPA1) and TRPV1, and stimulation of these receptors induces release of adrenomedullin (ADM) from the intestinal epithelium and calcitonin gene-related peptide (CGRP) from the nerve terminal in vascular smooth muscles, which is referred to as a vasodilator peptide hormone [7, 8]. Moreover, TJ-100 has an anti-inflammatory effect through the release of ADM and suppression of cycloxygenase-2 (COX-2) [9].

The clinical effects of some of these medicines have been accepted. In a previous study, the acceleration of gastrointestinal and colonic transit by TJ-100 was demonstrated in humans [10]. In addition, the acceleration of colonic motility was proven by colonoscopy in healthy volunteers [11]. Studies have shown that TJ-100 activated jejunal contraction in rabbit jejunum [12], spontaneous small intestinal activity in mice [13], and small intestinal motility in mice [14].

However, intestinal movement in itself has not been evaluated in humans because the use of radiography, fluoroscopy, and radioisotopes in healthy volunteers presents an ethical problem owing to ionizing radiation exposure. Therefore, an alternative imaging method for measuring intestinal movement is required for assessing pharmacological effects. Recently, cine magnetic resonance imaging (MRI) was shown to allow direct visualization of intestinal movement [15]. Previous studies have shown the clinical significance of cine MRI in the assessment of organic diseases, such as Crohn’s disease [16–18] and intra-abdominal adhesion [19–22]. In addition, cine MRI has been used to evaluate functional diseases, such as chronic intestinal pseudo-obstruction [23], pharmacological action associated with intestinal motility [24, 25], and intestinal motility after bariatric surgery [26]. In this way, cine MRI allows the evaluation of intestinal movement without ionizing radiation.

The effect of TJ-100 on small intestinal movements is unclear. Thus, the present study aimed to evaluate the effect of TJ-100 on small intestinal movements using cine MRI in healthy volunteers.

## Materials and methods

### Volunteers

This prospective double-blind study was approved by our institutional review board (approval number: 26–156), and written informed consent was obtained from all volunteers prior to their participation. In total, 10 healthy men (mean age, 39.1 [range, 25–53] years; mean body mass index, 23.2 [range, 20.3–26.7] kg/m^2^) were enrolled. The exclusion criteria were presence of ongoing gastrointestinal disease, history of abdominal surgery, and contraindications to MRI examination.

### Oral contrast agent administration

**Fig 1:**
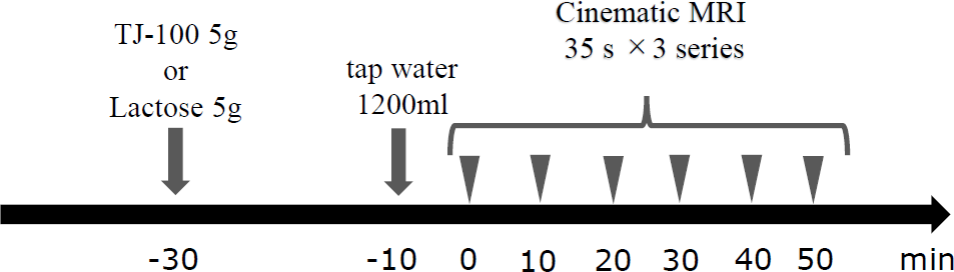
Examination protocol.

The volunteers fasted for 6 h without fluid intake for 2 h and were then administered TJ-100 (dai-kenchu-to, Tsumura, Tokyo, Japan) or lactose (lactose “Hoei,” Pfizer, Tokyo, Japan) at a dose of 5 g wrapped in a wafer, with eyes closed in order to block visual and gustatory information. A controller not involved in the evaluation of cine MRI prepared TJ-100 and lactose for the double-blind test. After 20 min, the volunteers drank 1200 mL of tap water to distend the small bowel and underwent MRI examinations (Fig 1). Each volunteer underwent two MRI examinations on separate days, and in the second examination, the other substance was administered (TJ-100 or lactose) after preparation as mentioned above. The interval between the two examinations was ≥1 week.

### MRI examination protocol

MRI examinations were performed using a 1.5-T MRI system (Signa HDx 1.5T; GE Healthcare, Milwaukee, WI, USA) with an 8-channel body array coil (GE Healthcare). The volunteers were placed in the prone position with breath held after inspiration during examination. Before real cine MRI, coronal images (FIESTA sequence: TR, 3.4 ms; TE, 1.1 ms; flip angle, 75°; slice thickness, 10 mm; matrix, 256 × 256; field of view, 450 mm) of the entire abdomen were obtained, and the best slice reflecting the maximum length of the small bowel was determined. A successive coronal scan consisting of 70 images in 35 s was obtained thrice in the respective selected plane. A steady-state free precession sequence (FIESTA sequence: TR, 3.4 ms; TE, 1.1 ms; flip angle, 75°; slice thickness, 10 mm; matrix, 256 × 256; field of view, 450 mm) was used for imaging. Imaging was performed in three series at 0, 10, 20, 30, 40, and 50 min after drinking tap water. Fig 1 shows the examination protocol.

### Imaging analysis

**Fig 2:**
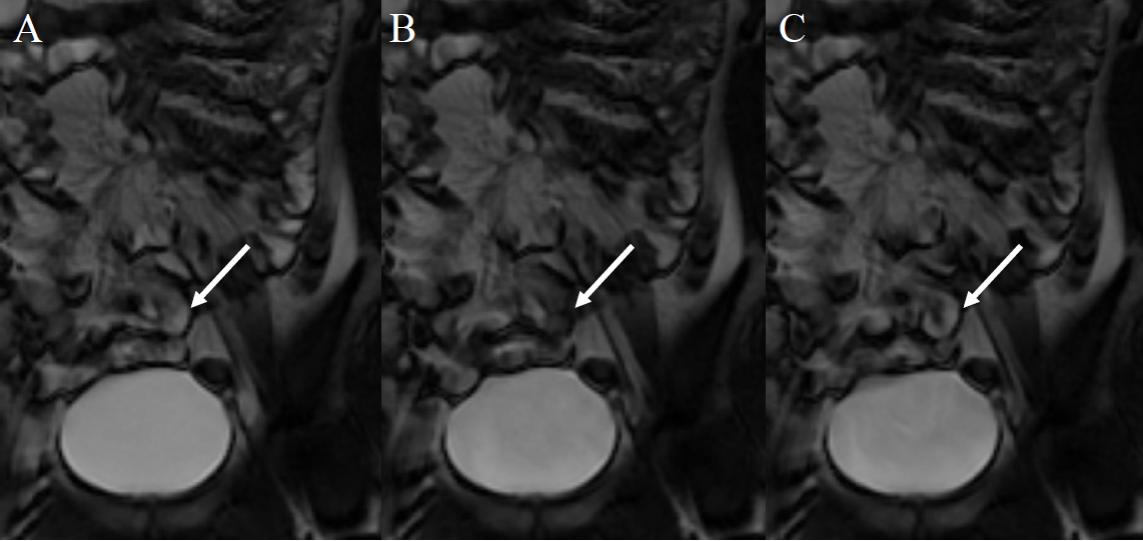
Evaluation of contraction frequency. The arrow indicates a representative ileum. The intestine is dilated (A), collapsed by contraction (B), and re-dilated (C). This cycle is counted as one contraction. Bowel contraction was judged as half contraction when the dilated intestine collapsed or the collapsed intestine dilated.

Image evaluations were performed by consensus between two radiologists with 9 and 32 years of experience reading gastrointestinal images, who were blinded to information about the administered substances (TJ-100 or lactose). A single loop in the left upper abdomen that stayed in a slice section during each scanning period and represented the patterns of contraction of other loops in the left upper abdomen was selected as a representative loop for the jejunum. In the same way, another loop in the right lower abdomen was selected as a representative loop for the ileum. Cine MRI images were analyzed for the frequency of bowel contraction (contraction frequency) of the representative loops in the jejunum and ileum in respective phases. The frequencies of contraction in the representative bowel loops were counted visually on cine MRI (Fig 2). Distended bowel loops were filled with fluid and demonstrated high intensity on MRI with our sequence, while collapsed bowel loops demonstrated low intensity that represented the intensity of the bowel wall, and the cycle of bowel contraction (dilation, collapse, and re-dilation) was reflected on cine MRI. Bowel contraction was judged as “full contraction = 1” when the entire cycle of contraction was confirmed, and it was judged as “half contraction = 0.5” when the dilated intestine collapsed or the collapsed intestine dilated.

**Fig 3:**
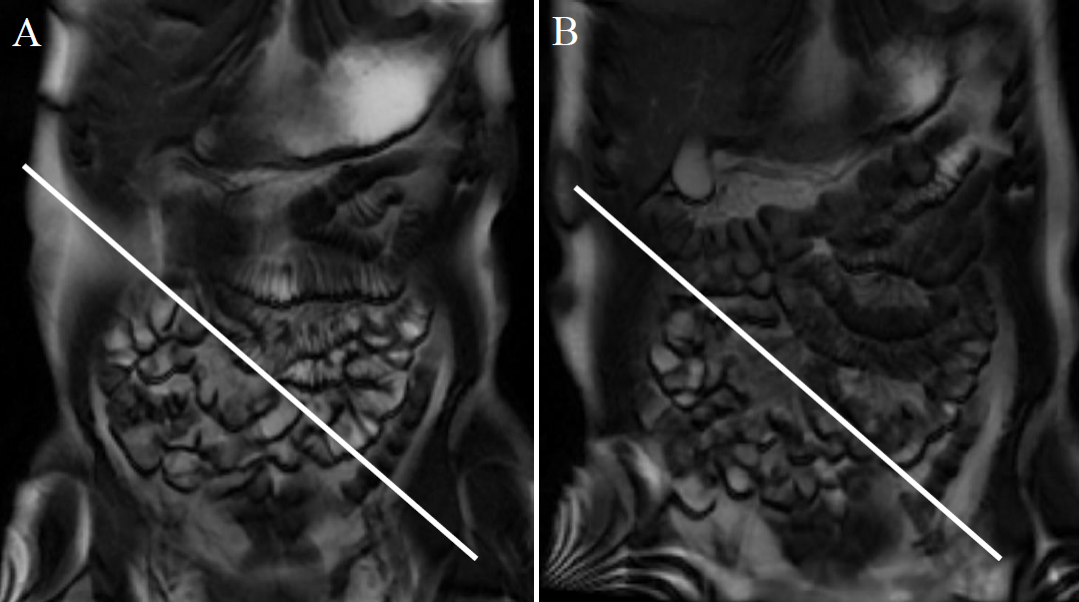
Evaluation of the bowel distention score. The small intestine separated by the white line in the left upper abdomen is the jejunum and by the white line in the right lower abdomen is the ileum. More than 70% of the jejunum and ileum is well filled with fluid, and the bowel distention score for both parts is “2” (A). Less than 30% of the jejunum is filled with a small amount of fluid, and the bowel distention score is “0” (B). Between 30% and 70% of the ileum is filled with a moderate amount of fluid, and the bowel distention score is “1” (B).

The degree of jejunal and ileal distention (distention score) was then assessed visually and scored using a 3-grade ranking. The distention score was judged as “0” when less than 30% of the loops of the jejunum or ileum were dilated, with fluid demonstrating high intensity on MRI. Similarly, the distention score was judged as “1” when between 30% and 70% of the loops of the jejunum or ileum were dilated and as “2” when more than 70% of the loops were dilated, with fluid demonstrating high intensity on MRI (Fig 3).

The results for the contraction frequency and distention score from three scans were added together in the respective phase.

### Statistical analysis

The mean contraction frequency and distention score in the respective phase was calculated by averaging the data of all volunteers. The Wilcoxon signed-rank test was used to compare the mean contraction frequency and distention score in the respective phase between the TJ-100 and lactose groups. The correlation between the added bowel contraction frequency and bowel distention score was evaluated using Spearman’s rank correlation coefficient. Statistical tests were performed using SPSS statistics 22 (IBM Corp., Armonk, NY, USA). A *P*-value <0.05 was considered to indicate a statistically significant difference.

## Results

Eight volunteers successfully completed all scans. Two volunteers in the lactose study were not able to undergo cine MRI at 30 min because of the urge to urinate. Therefore, statistical analysis at 30 min was performed without two volunteers.

### Bowel contraction frequency

**Fig 4:**
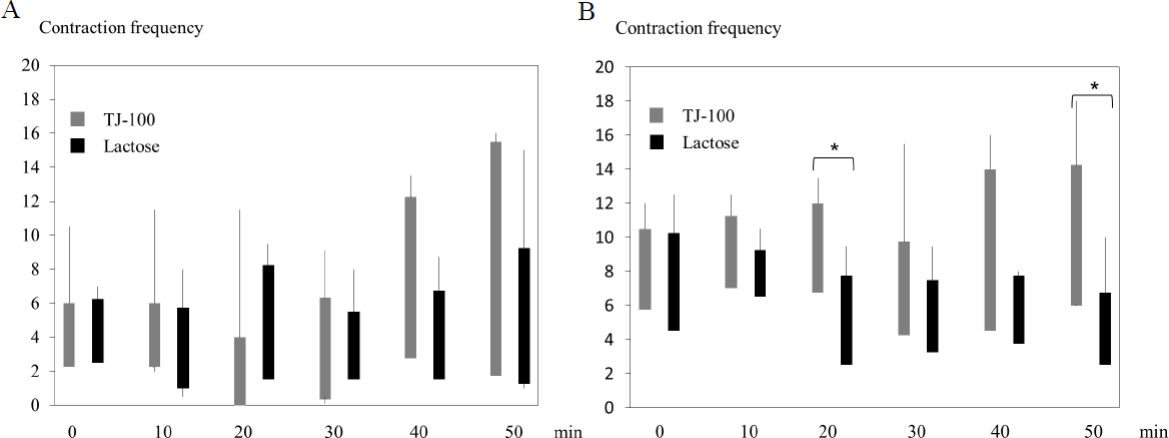
Contraction frequency. Bowel contraction tended to be more frequent in the jejunum in the TJ-100 group, without significance (A). Significant differences (*P < 0.05) are observed at 20 and 50 min in the ileum (B).

The total jejunal contraction frequencies were almost equal at 0 and 10 min and were higher at 20 and 30 min in the lactose group than in the TJ-100 group. In contrast, at 40 and 50 min, the total jejunal contraction frequencies were higher in the TJ-100 group than in the lactose group. There was no significant difference in jejunal contraction in any phase between the TJ-100 and lactose groups (Table 1 and Fig 4). The ileum contracted more frequently in all phases in the TJ-100 group than in the lactose group. There were significant differences in the contraction frequencies (*P* < 0.05) in the ileum at 20 and 50 min between the two groups (Table 1, Fig 4, and S1 and S2 Videos).

**Table 1:** Contraction frequency (number per 105 s).

Jejunum	TJ-100	Lactose	*P*-value
0 min	4.00 ± 2.85	3.90 ± 2.02	0.959
10 min	3.80 ± 3.30	2.80 ± 2.54	0.683
20 min	2.33 ± 3.54	4.70 ± 3.44	0.122
30 min	2.75 ± 3.29	3.19 ± 2.63	0.933
40 min	6.30 ± 4.84	3.6 ± 2.87	0.167
50 min	6.35 ± 6.24	3.60 ± 4.72	0.333
**Ileum**			
0 min	7.55 ± 2.58	6.85 ± 3.22	0.610
10 min	8.65 ± 2.70	7.80 ± 1.83	0.475
20 min	8.95 ± 2.88	4.80 ± 2.93	0.022*
30 min	7.05 ± 3.62	4.78 ± 2.75	0.271
40 min	8.10 ± 4.65	5.00 ± 2.49	0.137
50 min	9.45 ± 4.49	4.45 ± 2.65	0.014*

Data are presented as mean ± standard deviation (SD)

Significant differences (*P < 0.05) are observed at 20 and 50 min in the ileum

### Bowel distention score

**Fig 5:**
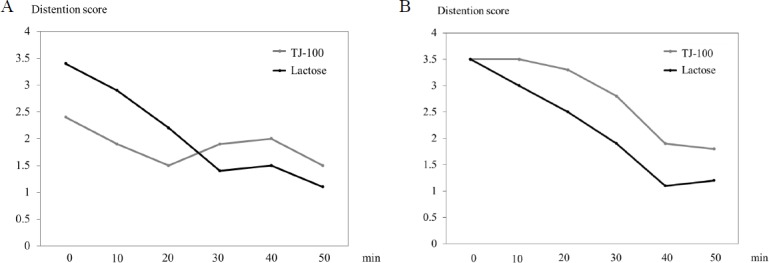
Distention scores. The score decreases gradually in both the jejunum (A) and ileum (B).

**Fig 6:**
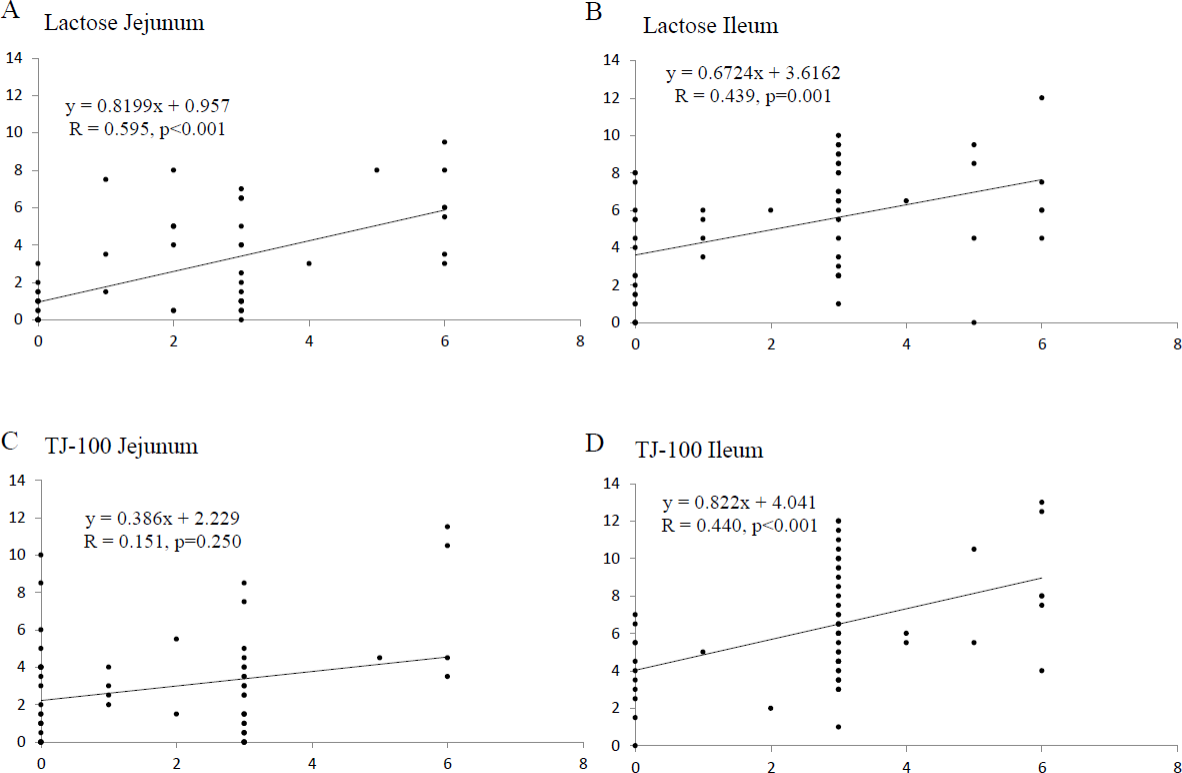
Interrelationship between the bowel contraction frequency and distention score. The points above the line show the relationship between the bowel contraction frequency (vertical axis) and distention score (horizontal axis) in the jejunum and ileum in all phases of respective volunteers. Spearman’s rank correlation coefficient shows positive correlations in the jejunum and ileum treated with lactose (A, B) and the ileum treated with TJ-100 (D).

The highest bowel distention score was observed at 0 min, and it gradually decreased with time in both the jejunum and ileum (Table 2 and Fig 5). There was no significant difference in the bowel distention scores in any phase between the TJ-100 and lactose groups. Fig 6 presents scatter plots with linear regression between the contraction frequency and distention score. Positive correlations were observed between the contraction frequency and bowel distention score in the jejunum and ileum. Spearman’s rank correlation coefficient showed statistical correlations for the jejunum in the lactose group (R = 0.595, p < 0.001) and TJ-100 group (R = 0.439, p < 0.001), and for the ileum in the TJ-100 group (R = 0.44, p < 0.001).

**Table 2:** Distention scores (number per 105 s).

Jejunum	TJ-100	Lactose	*P*-value
0 min	2.40 ± 2.01	3.40 ± 1.85	0.117
10 min	1.90 ± 1.81	2.90 ± 1.76	0.167
20 min	1.50 ± 2.01	2.20 ± 1.89	0.438
30 min	1.90 ± 1.92	1.40 ± 2.54	0.892
40 min	2.00 ± 1.34	1.50 ± 1.28	0.269
50 min	1.50 ± 1.50	1.10 ± 1.37	0.450
**Ileum**			
0 min	3.50 ± 1.02	3.50 ± 1.80	1.00
10 min	3.50 ± 1.12	3.00 ± 1.61	0.317
20 min	3.30 ± 1.62	2.50 ± 1.86	0.258
30 min	2.80 ± 2.14	1.90 ± 2.23	0.595
40 min	1.90 ± 1.37	1.10 ± 1.37	0.131
50 min	1.80 ± 1.47	1.20 ± 1.47	0.157

Data are presented as mean ± standard deviation (SD)

## Discussion

**Fig 7:**
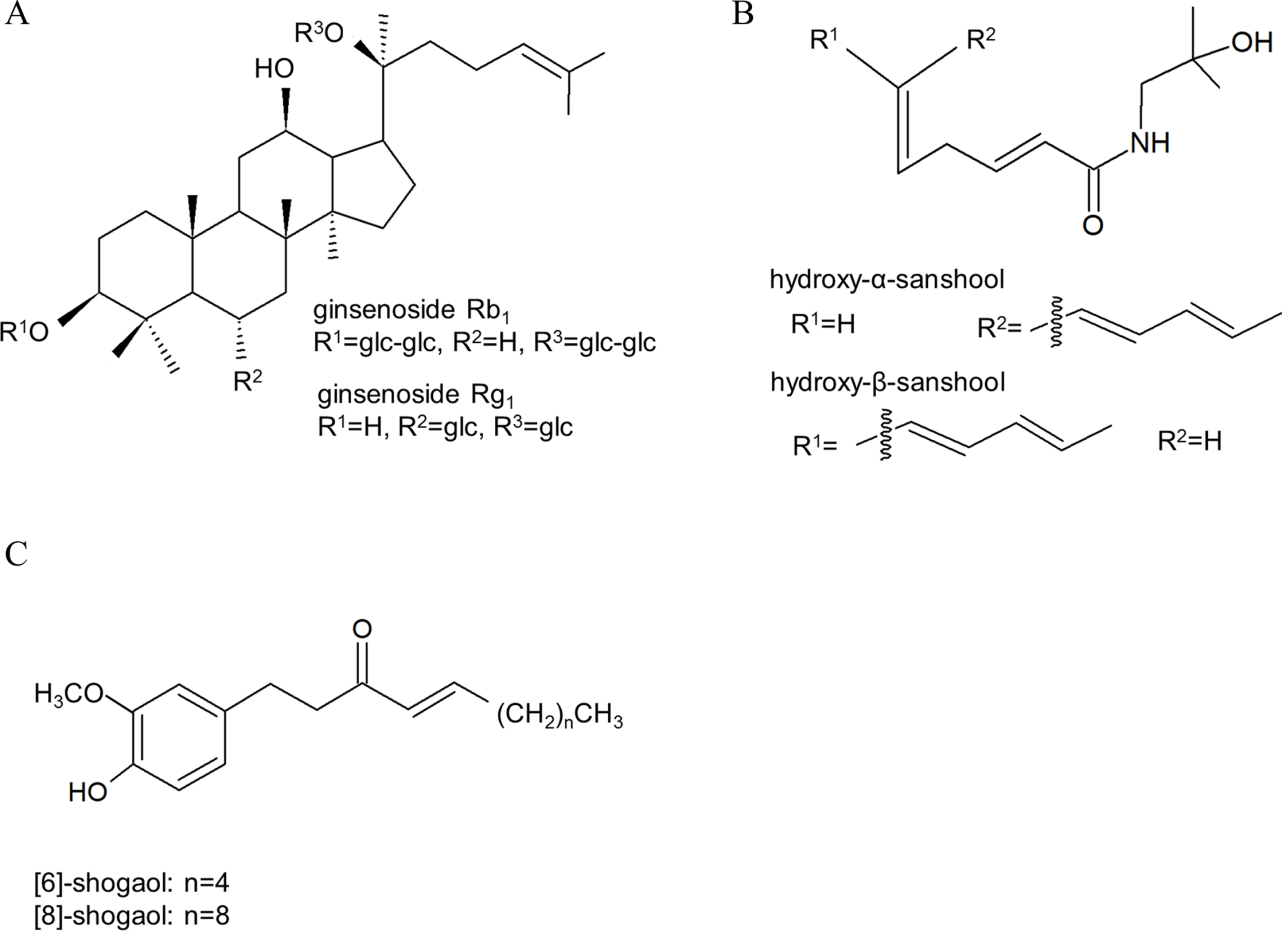
Major ingredients in TJ-100. A: ginsenoside, B: sanshool, C: shogaol.

TJ-100 is one of the medicines known to enhance bowel contraction and peristalsis, and it is often administered to patients whose bowel motility function is impaired, such as those who have undergone abdominal surgery [1]. In a previous study, the acceleration of gastrointestinal and colonic transit by TJ-100 was demonstrated in humans [10]. TJ-100 has been shown to include 44 ingredients by liquid chromatography–tandem mass spectrometry analysis [27]. Sanshool extracted from the zanthoxylum fruit, shogaols extracted from ginger, and ginsenosides extracted from ginseng are major ingredients in TJ-100 (Fig 7). Hydroxy-alpha-sanshool (HAS) has the highest plasma concentration among the ingredients in TJ-100, and it reaches the maximum concentration within 30 min after administration, with a median half-life of 1.6–1.7 h [28]. MRI in our study was performed 30–80 min after the administration of TJ-100, because the plasma concentration of HAS was higher than the effective level during that period.

In cine MRI, the steady-state free precession sequence has been widely applied, and it was used in this study. The sequence provides contiguous dynamic MRI images every 0.5 s, which enables cine MRI to demonstrate bowel contraction. Because the bowel should preferably be distended to a certain degree during imaging, various types of intraluminal fluids are administered transorally before MRI examinations. In previous reports, good bowel distention was obtained using Niflex (non-absorbable fluid by Ajinomoto, Tokyo, Japan), VoLumen containing 2.0% or 1.4% sorbitol [29], and 2.5% mannitol in tap water with or without 0.2% locust bean gum. Although tap water alone provides less bowel distention because of absorption during the examination, we used tap water in this study because we wanted to exclude any other factors that might influence bowel contraction. For the same reason, lactose was used as it is included in TJ-100 and has no pharmacological effects on bowel contraction.

Overall, both the jejunum and ileum tended to more frequently contract in volunteers treated with TJ-100 than in those treated with lactose, and the difference was more clear in the ileum. In the jejunum, relatively larger differences were observed at 40 and 50 min after completion of oral intake of the test fluid, although the differences did not reach significance. The reason why more frequent contractions were observed in volunteers treated with lactose at 20 min is unclear. The differences were more apparent in the ileum, and higher contraction frequencies continued at 50 min after completion of oral intake of the test fluid in volunteers treated with TJ 100, while contraction frequencies decreased after 20 min in those treated with lactose. Significantly more frequent contractions of the ileum were observed in volunteers treated with TJ-100 than in those treated with lactose at 20 and 50 min. The results may demonstrate higher and continuous stimulation of TJ-100 on bowel contraction in the ileum.

The collapsed bowel contracted less frequently than the distended ileum, and there was a linear relationship between the frequency of bowel contraction and the degree of bowel distention; i.e., greater bowel distention was associated with more frequent bowel contraction. This finding was consistent with the results of a previous study [15] and with the fact that bowel contraction is regulated by the volume and content in the bowel (i.e., fasting period, postprandial period, type of food, and amount of calories) and various factors, including intrinsic and extrinsic nerves and a variety of hormone and hormone-like substances [30, 31].

In the ileum, the interrelationship between bowel contraction frequency and distention score showed a positive correlation statistically in both volunteers treated with lactose and TJ-100. In the ileum, TJ-100 was associated with contraction frequencies independent from bowel distention because of comparisons under the same condition. On the other hand, in the jejunum, statistical analysis showed a positive correlation in volunteers treated with lactose, but no significant correlation in volunteers treated with TJ-100. Therefore, it was difficult to assess the pharmacologic effect of TJ-100 in the jejunum because the distention score was different between volunteers treated with lactose and those treated with TJ-100.

Postoperative bowel obstruction is a common complication of abdominal surgery, and sometimes recurrence can occur, which might require operation if strangulation is noted. TJ-100 has an anti-inflammatory effect through the release of ADM and suppression of COX-2, and it shortens the duration of intestinal transit, which prevents adhesion of the bowel, as seen in rats [9]. Previous articles have proved that TJ-100 can shorten the duration of intestinal transit [10]; however, intestinal movement in itself has not been evaluated in humans. The acceleration of bowel movement especially in the ileum observed in this study may possibly be one of the reasons for a reduction in the intestinal transit time with TJ-100, and accelerated bowel movement may contribute to a reduction in bowel adhesion as well. Our results may support the reasonable administration of TJ-100 in the postoperative phase for the prevention of adhesive bowel obstruction and symptoms caused by decreased propagative intestinal peristalsis. However, there has been no reported difference in the pharmacological mechanisms of TJ-100, including distribution in receptors of hormones related to motility between the jejunum and ileum, in our search, and therefore, the reasons for the difference in the pharmacological effects between the jejunum and ileum observed in this study are unclear and further investigations are required.

The present study has some limitations. First, the number of volunteers in the study was relatively small. Furthermore, imaging at 30 min was not performed in two volunteers. Second, bowel contraction was measured at certain selected loops. One in the left upper abdomen representing the jejunum and the other in the lower right abdomen representing the ileum. As reported by Menys et al. [32], who used MRI to quantify small bowel motility in normal volunteers, small bowel contraction may widely vary at each segment, with relatively poor repeatability over time. Thus, the measured loops in the present study may not have been good representatives of the jejunum and ileum, although we attempted to select loops representing the patterns of jejunal and ileal contraction blindly. This limitation may be overcome in future studies using different methods of assessment for bowel motility, such as the motility mapping method introduced by Hahnemann et al. [33]. Third, the cine MRI scan time for this study was 35 s because of the time restriction for breath holding at inspiration. Three scans were performed for the same slice in every phase; however, the scan time in each phase was still 105 s in total and could not cover all bowel motilities, such as that observed in physiological *in vitro* studies using an isolated small intestine. Finally, in this study, only jejunal and ileal contractions were compared between volunteers treated with TJ-100 and lactose. Neither the motility function of the stomach and colon nor the transit time was evaluated. Therefore, the results in this study may not be directly related to the effect of TJ-100 on clinical symptoms.

In conclusion, our results showed that cine MRI clearly assessed contraction and distention of the small intestine, which allowed motility functional assessment of the bowel. The finding that bowel contraction tended to be more frequent at 20 and 50 min in volunteers treated with TJ-100 suggests a positive pharmaceutical effect of TJ-100 on small bowel contraction.

## Supporting information

S1 Video Cine magnetic resonance imaging (MRI) performed in a volunteer treated with lactose at 50 min. Akinetic jejunum and ileum are observed on cine MRI.
(AVI)

S2 Video Cine magnetic resonance imaging (MRI) performed in a volunteer treated with TJ-100 at 50 min. This cine MRI was performed in the volunteer mentioned in S1 Video at 50 min. The ileal motility is greater with TJ-100 than with lactose (S1 Video).
(AVI)
